# More Than a Decade of Misdiagnosis of Alternating Hemiplegia of Childhood with Catastrophic Outcome

**DOI:** 10.1155/2017/5769837

**Published:** 2017-08-16

**Authors:** Hussein Algahtani, Bashair Ibrahim, Bader Shirah, Ahmad Aldarmahi, Ahad Abdullah

**Affiliations:** ^1^King Abdulaziz Medical City, King Saud bin Abdulaziz University for Health Sciences, Jeddah, Saudi Arabia; ^2^King Abdulaziz University, Jeddah, Saudi Arabia; ^3^King Abdullah International Medical Research Center, King Saud bin Abdulaziz University for Health Sciences, Jeddah, Saudi Arabia; ^4^King Saud bin Abdulaziz University for Health Sciences, Jeddah, Saudi Arabia; ^5^Batterjee Medical College, Jeddah, Saudi Arabia

## Abstract

Alternating hemiplegia of childhood (AHC) is a distinct clinical disorder characterized by recurrent episodes of hemiplegia, abnormal ocular movement, and progressive developmental delay. It is an extremely rare genetic disorder related to ATP1A3 gene mutations. In this paper, we present a case of AHC in which the diagnosis was missed for many years until severe hypoxic brain insult occurred from prolonged status epilepticus. Not only we are presenting an interesting clinical entity and radiological images, but also we are shedding the light on a rare genetic disease with catastrophic sequelae. The challenges in diagnosis and treatment lead to a poor outcome as seen in our case. Although early recognition and accurate diagnosis and treatment of the disease may not change the outcome, counseling of the family may change their expectation and reduce their frustration. Referral to a center with expertise in genetic disorders and access to genetic laboratories is of paramount importance in the diagnosis of this disease. Due to the rarity of this disease in Saudi Arabia, a genotype-phenotype correlation is not feasible.

## 1. Introduction

Alternating hemiplegia of childhood (AHC) is a distinct clinical disorder characterized by recurrent episodes of hemiplegia, abnormal ocular movement, and progressive developmental delay. It is an extremely rare genetic disorder related to ATP1A3 gene mutations with an estimated prevalence of 1/1,000,000 children. It is believed that this number could be an underestimate due to variability in clinical presentation, lack of knowledge about the disease, and lack of advancement in the diagnostic laboratory and radiologic test that will confirm the diagnosis [1]. AHC was first described in 1971 by Verret and Steele [2] who initially attributed the disorder to be a migrainous phenomenon. They reported 8 children with intermittent hemiparesis of varying severity with developmental delay, speech difficulties, and movement disorder. It was not until 1980 when Krägeloh and Aicardi [3] made the distinction between this disorder and migraine. Since then several case reports and large case series have been published which made the clinical spectrum of the disorder clearer. A thorough literature search yielded only one case study reported from Saudi Arabia [4]. In this paper, we present a case of AHC in which the diagnosis was missed for many years until severe hypoxic brain insult occurred from prolonged status epilepticus. We are not only presenting an interesting clinical entity and radiological images, but we are also shedding the light on a rare genetic disease with catastrophic sequelae.

## 2. Case Report

**Figure 1:**
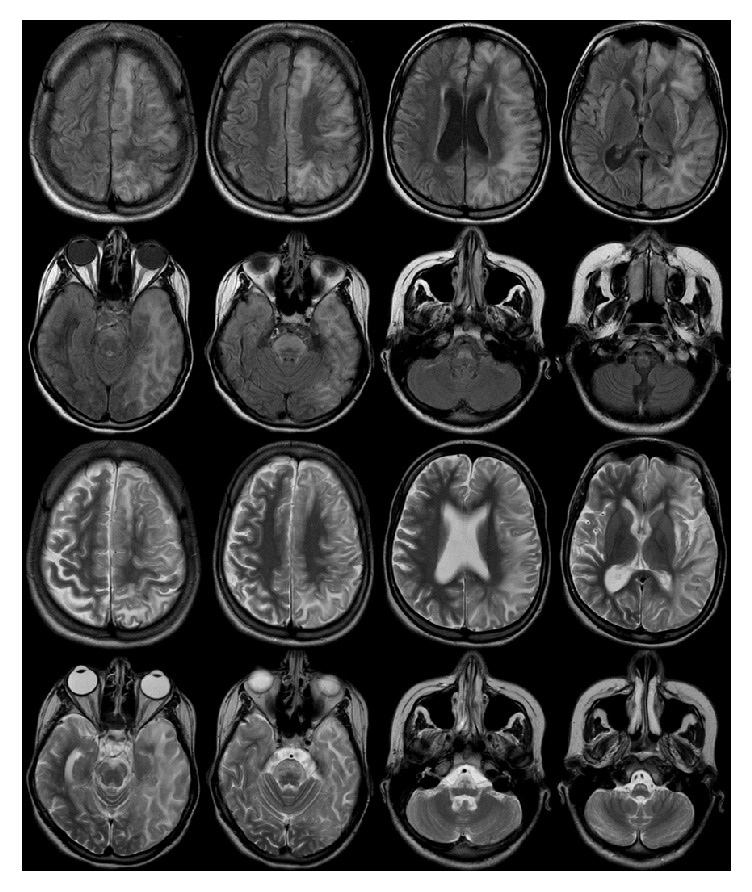
MRI of the brain showing evidence of diffuse asymmetrical, left more than right, cortical laminar necrosis secondary to refractory status epilepticus with anoxic brain damage. The images are FLAIR (upper two rows) and T2-weighted images (lower two rows). The changes are high signal intensity involving both supratentorial structures and brain stem.

A 15-year-old female was seen in neurology clinic after several episodes of transient paralysis that were started when she was one year old. Her mother stated that during these episodes she was fully conscious, but half of her body was paralyzed, and she had difficulty in speaking. The paralysis was characterized by recurrent episodes of transient hemiplegia alternating in laterality or affecting both sides simultaneously. There was no associated or preceding vomiting, fever, seizures, loss of consciousness, or headache. The episodes lasted from few hours to few days and recurred every 10–30 days without leaving any residual deficit. The paralysis disappeared after a good sleep and reappeared within half an hour after awakening. She was dysarthric when the episode was unilateral and mute when it was bilateral. The child became ataxic with deterioration of cognitive and intellectual functions after seven years of disease onset. She also lagged developmentally behind her chronological age. She was a product of a nonconsanguineous marriage at 39 weeks of gestation following a spontaneous vaginal delivery. The pregnancy and neonatal period were uneventful. There was no family history of a similar disease including migraine. Her neuroimaging was normal on several occasions. At the age of 14, she developed sudden onset of a partial motor seizure of the face with secondary generalization and status epilepticus. The seizures were difficult to control, lasted several hours, and necessitated an admission to the intensive care unit with intubation and mechanical ventilation. The final diagnosis was AHC with status epilepticus and catastrophic cortical necrosis due to severe hypoxic brain insult.  Figure 1 is showing her magnetic resonance imaging (MRI) few months following discharge from the hospital on multiple antiepileptic drugs. She was mute, quadriparetic, and psychotic. In a follow-up visit to the clinic, three years following discharge from the hospital, she showed some improvement in power and speech although continued to be fully dependent on the family for daily activities. She was also on multiple medications for her psychotic symptoms.

## 3. Discussion

**Figure 2:**
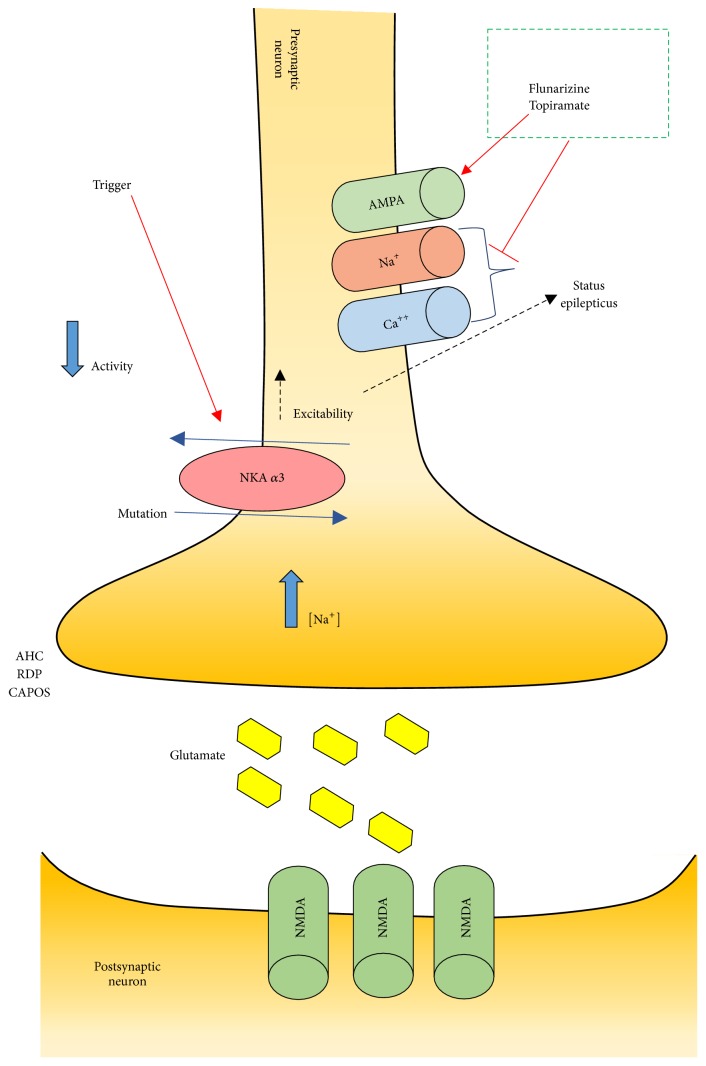
Schematic representation showing dysfunctional Na^+^/K^+^-ATPase pump activity due to ATP1A3 mutation in neurons affecting glutamatergic system activity. Mutation in ATP1A3 causes a decrease in Na^+^/K^+^-ATPase pump activity and an increase in intracellular Na^+^, which result in hyperexcitability that affects neuronal function. Flunarizine and topiramate are shown as treatment options for the conditions caused by this mutation.

AHC is a genetic disorder caused by mutations in the ATP1A3 gene which encodes an *α*-subunit (the *α*3-isoform) of the Na^+^/K^+^-ATPase pump. The Na^+^/K^+^-ATPase pump is partly responsible for establishing and maintaining electrochemical gradients of sodium and potassium ions across the plasma membrane of neurons. The *α*3-isoform is primarily found in the nervous system and is considered the most common form of *α*-subunits of the basal ganglia, hippocampus, and cerebellum. In addition to AHC, heterozygous mutations of the ATP1A3 gene have been reported in association with rapid-onset dystonia parkinsonism (RDP) and cerebellar ataxia, areflexia, pes cavus, optic atrophy, and sensorineural hearing loss (CAPOS) (Figure 2). Several cases with ATP1A3 gene mutation have been described with an intermediate AHC/RDP phenotype. ATP1A3 gene is responsible for 74% of AHC cases. However, other genes have been reported with similar clinical presentation including the ATP1A2 gene, GLUT-1 gene, and CACNA1A gene [5].

The spectrum of clinical symptoms of AHC is broad which makes the differential diagnosis difficult. The first sign of AHC typically arises prior to one year of age, often with evidence of mild developmental delay and abnormal eye movements. It is characterized by transient episodes of hemiplegia which last minutes to days and affect either one or both sides of the body. Brief episodes of monocular or binocular movements may occur including intermittent eye deviation, nystagmus, and dysconjugate gaze, which last for 1–3 minutes. The abnormal eye movements are most commonly unilateral and ipsilateral to the hemiplegia [6]. Motor attacks could be hemiplegia, quadriplegia, dystonia, or a combination of any of them. As a general rule, infants and young children have flaccid hemiplegia, and older children are more likely to have dystonic features. Dystonia may last from seconds to hours, and it is mostly unilateral. The attack onset is abrupt which makes dystonia often mistaken for a seizure and hemiplegia for a stroke. During a single hemiplegic attack, the intensity of weakness is fluctuating. During long attacks, hemiplegia may change from one side to the other, or both sides may be affected. The arm is mostly weaker than the leg, and walking may not be impaired. Hemiplegia ceases during sleep and reappears on awakening but not immediately. Deterioration of consciousness was not associated with episodes of hemiplegia. Dystonic episodes may primarily affect the limbs on one side, causing hemidystonia, or affect the trunk, causing opisthotonic posturing. Headaches could occur at the onset of an attack but not after it. Writhing movements that suggest choreoathetosis could also be an associated feature. Mental slowing occurs early in the disease course, while mental regression and persistent neurological abnormalities could follow late in the course of the disease [7].

The evaluation of AHC is focused on excluding other serious or treatable causes. MRI, magnetic resonance angiography (MRA), and magnetic resonance spectroscopy should be performed to exclude structural, vascular, and metabolic disorders including mitochondrial myopathy, encephalopathy, lactic acidosis, and stroke (MELAS) and pyruvate dehydrogenase deficiency. Other differential diagnoses include different subgroups of stroke, epilepsy syndromes, and different types of migraine. Results of neuroradiological tests in AHC cases are usually normal. However, in the severely affected or older patients, changes may include cerebellar atrophy, polymicrogyria, syringomyelia, or hippocampal pathology. Other testing may include electroencephalogram (EEG), metabolic screening with urine organic acids, quantitative serum and cerebrospinal fluid (CSF) amino acids, acylcarnitine, lactate/pyruvate (serum and CSF), hypercoagulable studies, erythrocyte sedimentation rate, and transferrin isoelectric focusing. Genetic testing of the ATP1A3 gene is also important for AHC confirmation [8].

AHC treatment can be divided into acute management of the attacks and episode prophylaxis. Acute management is focused towards removing known triggers and early sleep facilitation. The use of buccal midazolam or rectal diazepam has been advocated by some authors to provide quick sedation. Episode prophylaxis is focused towards avoiding known triggers and long-term drug treatment. A variety of medications have been proposed for the treatment of AHC, but calcium channel blockers are the most effective. The most common calcium channel blocker used is flunarizine in a dose of 5 to 20 mg per day. Flunarizine has been reported to reduce the frequency and severity of attacks, but not to completely stop them, and it is considered the drug of choice [9]. Other proposed treatments include beta blockers, anticonvulsants, methysergide, amantadine, aripiprazole, and haloperidol. Antiepileptic drugs are effective in treating seizures only. Topiramate has been reported to positively influence the severity in some patients with AHC [10]. A recent article reporting the use of adenosine-5′-triphosphate orally with 2-year follow-up demonstrated promising and successful results [11]. In addition, some reports supported the use of a ketogenic diet in patients with AHC [12, 13].

The long-term outcome of patients with AHC is generally poor due to the associated developmental delays and gradual deterioration after severe attacks. The clinical course of AHC is more severe in sporadic cases than in familial ones. Prognosis is greatly influenced by the age of onset, especially early occurrence of hemiplegic spells. Children with neonatal-onset manifestation usually suffer from severe developmental delay. Recurrent convulsive status epilepticus leads to deterioration of psychomotor development. In some children, motor dysfunctions caused wheelchair-dependency, but others were able to have an independent life in adulthood. As patients become older, hemiplegic spells and abnormal ocular movements become less common and hypotonia less severe [7].

## 4. Conclusion

Since the original description of AHC, many endeavours have been made to understand the pathophysiology of the disease which resulted in linking the disease with mutations in the gene ATP1A3. Despite this substantial progress in the understanding of the disease, no curative treatment has been discovered, and the disease continues to be challenging to treat. All the current treatments are focused on reducing the frequency, duration, and severity of AHC episodes. The challenges in diagnosis and treatment lead to a poor outcome as seen in our case. Although early recognition and accurate diagnosis and treatment of the disease may not change the outcome, counseling of the family may change their expectation and reduce their frustration. Referral to a center with expertise in genetic disorders and access to genetic laboratories is of paramount importance in the diagnosis of this disease. Due to the rarity of this disease in Saudi Arabia, a genotype-phenotype correlation is not feasible. The complexity and severity of this disorder make more research crucial to find the curative therapy and further understand the disease.
